# Antimicrobial management and appropriateness of treatment of urinary tract infection in general practice in Ireland

**DOI:** 10.1186/1471-2296-12-108

**Published:** 2011-10-03

**Authors:** Akke Vellinga, Martin Cormican, Belinda Hanahoe, Kathleen Bennett, Andrew W Murphy

**Affiliations:** 1 Discipline of General Practice, School of Medicine, NUI Galway, Ireland; 2 Department of Medical Microbiology, University Hospital, Galway, Ireland; 3 Centre for Health from Environment, Ryan Institute, NUI Galway, Ireland; 4 Department of Pharmacology & Therapeutics, Trinity Centre for Health Sciences, Dublin, Ireland

**Keywords:** management, UTI, antimicrobial prescribing

## Abstract

**Background:**

Urinary tract infections (UTIs) are the second most common bacterial infections in general practice and a frequent indication for prescription of antimicrobials. Increasing concern about the association between the use of antimicrobials and acquired antimicrobial resistance has highlighted the need for rational pharmacotherapy of common infections in general practice.

**Methods:**

Management of urinary tract infections in general practice was studied prospectively over 8 weeks. Patients presenting with suspected UTI submitted a urine sample and were enrolled with an opt-out methodology. Data were collected on demographic variables, previous antimicrobial use and urine samples. Appropriateness of different treatment scenarios was assessed by comparing treatment with the laboratory report of the urine sample.

**Results:**

A total of 22 practices participated in the study and included 866 patients. Bacteriuria was established for 21% of the patients, pyuria without bacteriuria for 9% and 70% showed no laboratory evidence of UTI. An antimicrobial agent was prescribed to 56% (481) of the patients, of whom 33% had an isolate, 11% with pyuria only and 56% without laboratory evidence of UTI. When taking all patients into account, 14% patients had an isolate identified and were prescribed an antimicrobial to which the isolate was susceptible. The agents most commonly prescribed for UTI were co-amoxyclav (33%), trimethoprim (26%) and fluoroquinolones (17%). Variation between practices in antimicrobial prescribing as well as in their preference for certain antimicrobials, was observed. Treatment as prescribed by the GP was interpreted as appropriate for 55% of the patients. Three different treatment scenarios were simulated, i.e. if all patients who received an antimicrobial were treated with nitrofurantoin, trimethoprim or ciprofloxacin only. Treatment as prescribed by the GP was no more effective than treatment with nitrofurantoin for all patients given an antimicrobial or treatment with ciprofloxacin in all patients. Prescribing cost was lower for nitrofurantoin. Empirical treatment of all patients with trimethoprim only was less effective due to the higher resistance levels.

**Conclusions:**

There appears to be considerable scope to reduce the frequency and increase the quality of antimicrobial prescribing for patients with suspected UTI.

## Background

Urinary tract infections (UTIs) are the second most common bacterial infections in general practice and a frequent indication for prescription of antimicrobials [1]. Antimicrobial resistance in urinary pathogens, particularly *E.coli*, is directly associated with prescribing in primary care [2]. Increasing concern about the association between the use of antimicrobials and acquired antimicrobial resistance has highlighted the need for rational pharmacotherapy of common infections in general practice [3].

Diagnosis of UTI often requires laboratory examination of a urine sample in addition to clinical evaluation. Although many guidelines indicate that the culture of urine is not required in most cases of uncomplicated cystitis, the laboratory in this region accepts all such requests from the GP and many GPs choose to send samples on all patients with suspected UTI [4,5]. For patients with symptomatic UTI empiric antimicrobial treatment is generally recommended while culture and susceptibility results are pending. Pyuria is an expected accompaniment of significant bacteriuria. The absence of pyuria is considered useful in excluding UTI [6]. However, pyuria accompanying asymptomatic bacteriuria is not of itself an indication for antimicrobial treatment [7]. Trimethoprim and nitrofurantoin are the agents generally recommended for empiric therapy of uncomplicated UTI although acquired resistance to trimethoprim in *E.coli *may limit the use of this agent for empiric treatment in many areas [7-9].

Even though UTI is a very common diagnosis, management of this condition is not consistent in general practice [3,10]. The aim of our study was to describe the current management of UTI in general practice including the evaluation of appropriateness of the antimicrobial treatment in relation to the laboratory results.

## Methods

The mainly rural population served by the laboratory is approximately 230,000 individuals within 72 general practices. The laboratory of the Galway University Hospitals (GUH) analyses all urine samples from the practices in this region. The study required that practices submitted samples for every patient with suspected UTI on clinical grounds. In order to minimise the study impact on laboratory workload and established GP practice, practices with already high numbers of submitted samples were invited to participate. Practices were ranked according to the number of submitted urine samples in 2007 and based on samples size calculations, economic and time constraints, the 25 highest ranking practices were invited to participate in the study. Of these, 22 practices participated, two practices were not included as they did not have computerised records and one practice declined (they never participated in research).

From the 14th of September to the 9th of November 2009 (8 weeks) practices were requested to send a urine sample from all adult patients presenting with symptoms of urinary tract infection. Notices outlining the study were put up in all practice waiting and consultation rooms. A website was set up with information on antimicrobial resistance and detailed information on the study (http://www.antibiotics.nuigalway.ie). All adult patients (≥18 years of age) from whom a urine sample was received in the laboratory were informed of the study by a letter explaining the study. Patients could opt-out of the study by returning the included (freepost) opt-out form, by filling in the opt-out form on the website or by telephone. All practices were visited and the charts of all participating patients were viewed for information on demographic variables and all antimicrobial prescribing in the year previous to the urine samples was recorded. Patients with indwelling catheters and pregnant women were excluded from the study.

Ethical approval was obtained from the Research Ethics Committee of the Irish College of General Practitioners. More detail on the ethical approval, the selection of practices as well as on the inclusion of patients has been described in a previous paper [11].

The receiving laboratory is accredited to the ISO 15189 quality standard. Laboratory diagnosis was based on microscopy and semi quantitative culture of a urine sample. Pyuria was defined as the presence of increased leukocytes (greater than 20 white cells/μl) in the urine. For the purpose of this study, bacteriological confirmation of UTI was defined as a pure or predominant growth of relevant organisms at the level of 10^5 ^colony forming units (cfu) per ml [12]. Antimicrobial susceptibility testing was performed in accordance with the disk diffusion method of the Clinical and Laboratory Standards Institute (CLSI) [13]. For further statistical analysis, samples with intermediate resistance were categorised as resistant.

Antimicrobial prescriptions were recorded according to the major groups: penicillins, β-lactam/β-lactamase inhibitor combinations, cephalosporins, trimethoprim, fluoroquinolones (including nalidixic acid and fluoroquinolones such as ciprofloxacin), tetracycline, nitrofurantoin and macrolides. Information on previous urine samples was also included. Other variables available for patients were age, gender, number of visits in the previous year (more or less than 10). Medical card eligibility depends on income and age and can be interpreted as a proxy measure of socio-economic status. At the time of the study, about 30% of the population was eligible for a medical card including all pensioners over the age of 70 years. Medical card patients have free medical care and medication while other (private) patients pay for both.

For each patient the first visit during the study period at which a urine sample was obtained, was used as the index visit. The antimicrobial therapy prescribed in a period of 7 days around the date of the sample, was identified to be the medication given for this episode, with the closest prescription to the date of urine collection regarded as the initial prescription.

Appropriateness of different treatment approaches was assessed by evaluating the treatment prescribed by the GP with the subsequent laboratory report of the urine sample. Overall, antimicrobial prescription for patients without bacteriological confirmation of infection was considered inappropriate. The prescription of an antimicrobial to which the cultured organism was resistant was also considered inappropriate. Appropriate treatment included not prescribing an antimicrobial to patients with a negative culture and treating patients with an isolate susceptible to the antimicrobial prescribed. The antimicrobial prescriptions for patients with an isolate which was not *E.coli *were individually assessed. A simulation of different treatment options, i.e. if all patients who received an antimicrobial were treated with nitrofurantoin, trimethoprim or ciprofloxacin, is also presented. Based on the relative numbers of *E.coli *in each treatment group, an additional analysis was performed to assess at what trimethoprim resistance level the trimethoprim scenario would reach similar levels of appropriate treatment as nitrofurantoin and fluoroquinolones. The price of treatment was calculated from the average price of all the prescriptions of these practices for this antimicrobial group. Prices were according to the manufacturing cost of the medicine as recorded in the HSE-PCRS (year 2010).

Statistical analysis was performed with SPSS (version 18.0) for univariate comparisons and WinPepi for the comparison of proportions [14].

## Results

A total of 1361 urine samples were submitted from 1163 patients of whom 145 (12.5%) opted out of the study and 152 (13%) patients did not meet the eligibility criteria. Of the 866 eligible patients the mean age was 52.4 years (95% CI 51.0-53.8, ranging from 18-100) and 77.9% were females. For 183 out of the 866 patients UTI was confirmed by culture (21%), mainly *E.coli *(147 or 80.3%). Other organisms identified were *Proteus spp. (9)*, other *Enterobacteriaceae *(8) *Staphylocccus saprophyticus (6)*, *Enteroccus *spp. (5) and eight other species. Pyuria was detected in 76 (8.8%) patients and 607 (70.1%) patients showed no laboratory evidence of UTI.

An antimicrobial was prescribed to 56% of the patients (481). An overview of antimicrobial prescribing is shown in table 1. Co-amoxyclav (33.1%) and trimethoprim (26.0%) were most often prescribed, fluoroquinolones represented 17% of the prescriptions and nitrofurantoin nearly 12%. In two cases it was not possible to determine from the record which antimicrobial agent was prescribed. More than half of the antimicrobials prescribed (55.7%) were for patients with no laboratory evidence of UTI and 11% were for patients with pyuria only. In total 179 of the patients (37% of all prescriptions) received a recommended first line antimicrobial (nitrofurantoin or trimethoprim).

**Table 1:** Overview of antimicrobial therapy at the index visit according to the microscopy result of the urine sample

	Community resistance level in 2009 (*E.coli *only)		no organism, no pyuria	Pyuria	organism	Total
Co-amoxyclav	30.5%	N	87	21	51	159

		% of total	18.1%	4.4%	10.6%	33.1%

		% within	54.7%	13.2%	32.1%	

						

Ampicillin	68.1%	N	18	1	8	27

		% of total	3.7%	0.2%	1.7%	5.6%

		% within	66.6%	3.7%	29.6%	

						

Trimethoprim	30.5%	N	60	15	50	125

		% of total	12.5%	3.1%	10.4%	26.0%

		% within	48.0%	12.0%	40.0%	

						

Fluoroquinolone	8.3%	N	54	8	20	82

		% of total	11.2%	1.7%	4.2%	17.0%

		% within	65.9%	9.8%	24.4%	

						

Nitrofurantoin	4.1%	N	29	6	22	57

		% of total	6.0%	1.2%	4.6%	11.9%

		% within	50.9%	10.5%	38.6%	

						

Other		N	20	4	7	31

		% of total	4.2%	0.8%	1.5%	6.4%

		% within	64.5%	12.9%	22.6%	

						

Total		N	268	55	158	481

		% of total	55.7%	11.4%	32.8%	100.0%

The number (N) and percentage (% within) of each antimicrobial prescribed is given for patients with no organism or pyuria was identified in the urine sample, when only pyuria was detected and when an organism was detected. The overall percentage of each antimicrobial prescribed (% of total) is also shown. For reference, the community antimicrobial resistance levels of all *E.coli *from urinary samples (no duplicates) analysed in the laboratory of the Galway University Hospitals during 2009 is presented in the first column.

**Figure 1:**
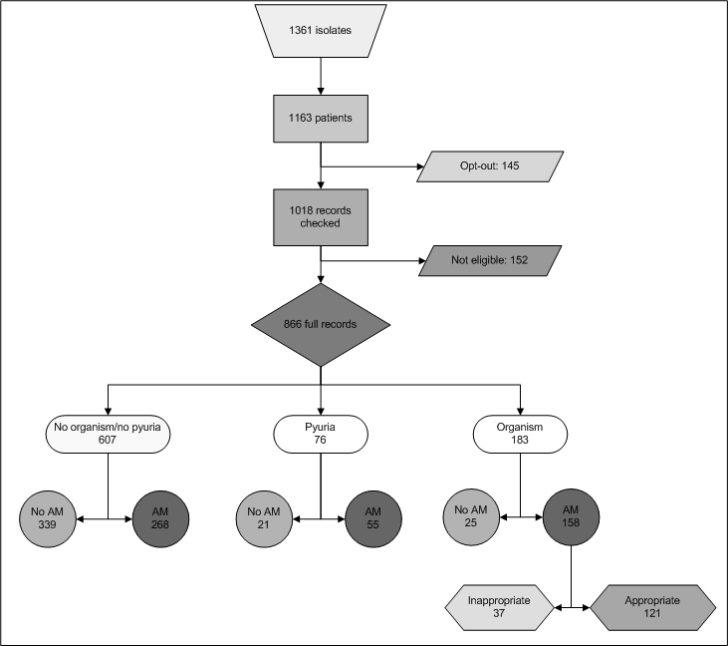
**Flow chart (AM is AntiMicrobial therapy) of the study population and management of UTI in general practice**.

Of the 158 patients with bacteriological confirmation of infection, the antimicrobial susceptibility was known and compared with the antimicrobial therapy prescribed. Co-amoxyclav was prescribed 51 times but for 10 of those the isolated organisms was resistant (20%) to co-amoxyclav, for ampicillin 6 out of 8 prescriptions (75%) were for patients with ampicillin resistant organisms, for trimethoprim 18 out of 50 (36%), for ciprofloxacin 2 out of the 20 (10%) and for nitrofurantoin 2 out of 22 prescriptions (9%) were prescribed for an infection with a nitrofurantoin resistant organism. No nitrofurantoin was prescribed for *Proteus spp*. In total 37 out of 158 patients (23%) were prescribed an agent to which the isolate cultured was resistant. When taking all records into account, 121 out of 866 patients (14.0%) had a laboratory confirmed UTI and were prescribed an appropriate antimicrobial to which the isolate was susceptible. A flow chart of the isolates included in the study is shown in Figure 1.

### Practice differences

**Figure 2:**
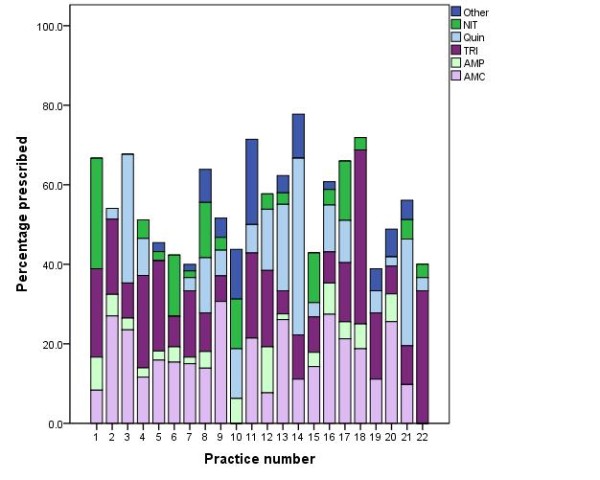
**Overview of total and specific antimicrobial prescribing by practice**.

Practices showed preferences for certain antimicrobials and prescribing differed considerably between practices. An overview of practice prescribing is shown in Figure 2. The percentage of the patients receiving any antimicrobial therapy ranged between 39% and 78% between practices. Some practices mainly prescribed trimethoprim and nitrofurantoin, according to the recommended first line treatment of UTI; practice 18, practice 22 and practice 1, while other practices predominantly prescribe fluoroquinolones (practice 14).

Participating practices were compared with non-participating practices for the available variables from the laboratory (percentage female and mean age of patients, percentage *E.coli *positive samples and percentage resistance for each antimicrobial) and showed no significant differences (t-test, comparing means, results not shown).

### Appropriateness of treatment

**Figure 3:**
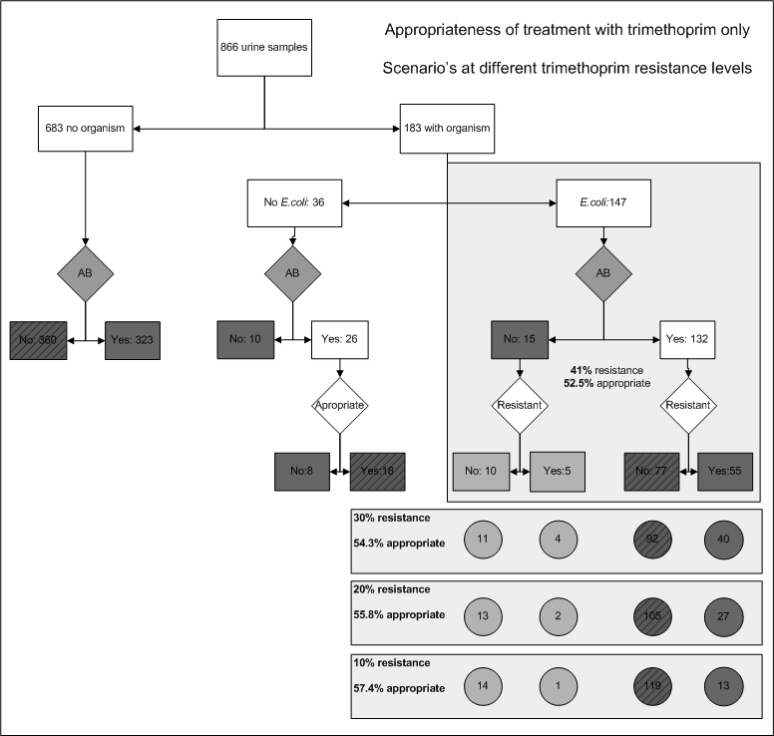
**Appropriateness of treatment of UTI with trimethoprim only (appropriately treated patients marked by diagonal pattern)**.

An antimicrobial treatment was prescribed to 481 of the 866 patients included in this study, the two unknown antimicrobial therapies were interpreted as appropriate. The appropriateness of treatment was evaluated against the laboratory report on the urine sample and interpreted as appropriate for 55% of the patients (table 2). Additionally, the appropriateness of three specific scenarios was assessed; all antimicrobial prescriptions were nitrofurantoin (scenario 1), trimethoprim (scenario 2) or ciprofloxacin (scenario 3). The analyses of these scenarios showed that treatment as prescribed by the GP, the nitrofurantoin only and the ciprofloxacin only scenario reached similar levels of appropriately treated patients (Comparison of proportions of two independent groups [14], no significant difference). The medication cost was lower for the nitrofurantoin only scenario, the ciprofloxacin only scenario resulted in the highest medication cost and the actual GP prescribing was intermediate. Additional use of urine dipstick to exclude prescribing of nitrofurantoin for patients with alkaline urine (often resulting from an infection with *Proteus spp*. for which nitrofurantoin would be considered inappropriate treatment) could further increase the appropriateness of treatment of UTI with nitrofurantoin ([15]. Empirical treatment of all patients with trimethoprim only was less often appropriate due to the higher resistance levels. The additional analysis of the trimethoprim only scenario at different trimethoprim resistance levels resulted in 57.4% of patients appropriately treated when at resistance levels for trimethoprim of 10% (Figure 3) but fall below that with increasing trimethoprim resistance levels.

**Table 2:** Overview of the appropriateness of treatment prescribed by the GP and is cost

Treatment option	Appropriately treated patients N (%)	Inappropriately treated patients N (%)	Cost of medication
As prescribed by GP	478 (55.2)	388 (44.8)	€ 3,685 *

Empiric treatment with nitrofurantoin only	498 (57.5)	368 (42.5)	€ 2,222

Empiric treatment with trimethoprim only	455 (52.5)	411 (47.5)	€ 2,001

Empiric treatment with fluoroquinolones only	499 (57.6)	367 (42.4)	€ 7,691

Three treatment scenarios and their cost are also presented, i.e. if an antimicrobial was prescribed by the GP, what if this prescription was only nitrofurantoin, trimethoprim or fluoroquinolone.

*2 unknown prescriptions not included

## Discussion

### Overview of the results

Overall, only 1 in 5 urine samples of patients with suspected UTI have bacteriological confirmation of UTI by the criteria applied in this study. A high proportion of patients (481 or 56%) were treated with antimicrobials. Of these 481 patients, 56% (268) had no laboratory evidence of UTI and a further 11% (55) of these patients have pyuria but no significant bacteriuria. A number of these prescriptions were not appropriate because the organism was resistant or because the treatment was not appropriate for the infection with the organism identified in the urine culture. Recommended first line treatment was prescribed for just 38% of patients (trimethoprim or nitrofurantoin). Treatment of UTI in general practice shows important variation between practices and clear practice preferences for certain antimicrobials. This was most striking for fluoroquinolones, which is the prescription of first choice in some practices, and which was prescribed to 17% of all patients in the study.

Of all patients in the study, 56% received treatment that was classified as appropriate; no treatment when this was not necessary (42%) or appropriate treatment in case of a laboratory confirmed UTI (14%). If all antimicrobial prescriptions by the GP were for nitrofurantoin, 57% of the treatments would have classified as appropriate with a total drug cost that would have been substantially lower than the total cost of the antimicrobial treatments actually prescribed by the GP. While there are certain patients for whom nitrofurantoin is not appropriate [7,8], this finding supports recommendations for the use of nitrofurantoin as the preferred agent for empiric therapy in the absence of a specific contraindication. Even though fluoroquinolones also reach this higher level of appropriateness, the cost of treatment with fluoroquinolones is nearly 3.5 times higher compared to the nitrofurantoin treatment. In addition, there are specific concerns related to the widespread use of fluoroquinolone agents [7,16]. The proportion of *E.coli *bloodstream isolates resistant to ciprofloxacin has increased considerably in Ireland and other European countries in recent years [17,18]. Blood stream infection with fluoroquinolone resistant *E. coli *is associated with an increased risk of mortality, most likely due to the delayed adequate antimicrobial therapy [19,20].

### Strengths and limitations

The high level of participation of patients due to the opt-out method of recruitment ensures the reliability of the study results. This together with the representativeness of the participating practices as well as the similarity between the urine submission patterns before and during the study period [11] results in a comprehensive overview of current management of UTI in Irish general practice.

To assess the appropriateness of therapy, a period of seven days around the submission of the urine sample was used. Empirical therapy is initiated before the causative pathogen is identified and the prescription of antimicrobial treatment aims to be active against the most likely pathogens, taking into account local resistance profiles while not increasing the potential impact on resistance levels [21]. As it was possible to have the laboratory results after 3 days and antimicrobial treatment could have been based on these results, patients with an antimicrobial treatment prescribed between 4 and 7 days after submission were identified. A total of 23 patients received antimicrobial during this window (out of a total of 481 who received an antimicrobial). Of these, no organisms was identified for 14 patients even though they obtained an antimicrobial prescription 4 days after their urine sample was taken, 8 received an appropriate antimicrobial and one patient's treatment was changed due to the resistance of the isolate. In addition to this patient, only one other patient had their prescription changed due to the *E.coli *being resistant to the first treatment.

The categorisation of empirical treatment of UTI as appropriate or not has limitations as it does not include a validation against the guidelines for first-line antimicrobials [7,8] and considers appropriate the prescription of agents with a wider spectrum of activity than necessary (ciprofloxacin and co-amoxyclav). To address the limitation of this approach data on the extent of prescribing of individual antimicrobial agents were included to show the extent of the overuse of ciprofloxacin and co-amoxyclav. The gap between recommended practice (guidelines) and actual clinical practice is common in primary and hospital care [22,23]. Additional qualitative work is necessary to study and describe both why this is happening and what is required to change such behaviour.

For bacteriuria a threshold of 10^5^/ml was used in this study and for pyuria a threshold ≥ 20 leucocytes/ml was defined. Pyuria alone is not a reliable indicator of urinary tract infection as it may be present as a result of other conditions [12,24]. However, as lower thresholds for bacteriuria can be used and pyuria in itself may be a result of antimicrobial treatment of UTI before collection of the sample, the appropriateness of the treatment might be underestimated. For the comparison of the treatments this difference would be expected to be the same for all organisms and treatment options and would not affect the conclusions.

### How this compares

The high number of participants in this study shows the success of the op-out methodology. A paper from Germany on management of UTI in female patients [25], enrolled 585 patients with suspected UTI from 36 practices over a period of 4 months while Fahy *et al. *[26] enrolled 160 patients from 8 practices in Bristol over a 4 month period. An observational study recruited 288 patients from 9 practices and required the GP to enrol patients and patients to fill out a questionnaire [27] and obtained 60% response and 39% subsequent participation. A prospective study with two recruitment arms obtained 66% participation for patients approached within the healthcare facility and 41% participation in a random sample of non attending patients [28]. In a spotter practice model, clinicians from three general practices were asked to submit mid-stream urine samples from all patients presenting with symptoms suggestive of UTI [29]. The percentage of patients with significant bacteriuria (according to the same laboratory definition as in our study) was 26%. Additional information from a sentinel practice group showed significant growth in 28% of the urines samples received [30]. Our results with a high inclusion rate (866 patients over 2 months) and 21% positive urine samples compares favourably. The high inclusion is partially due to the fact that the GP's in our study were not requested to enrol the patients and neither the patient nor GP were requested to provide additional information. This additional information is available from the other studies which can be seen as the trade off between more detailed patient information and a more representative population.

It is clear from our results that decisions on empiric prescriptions of antimicrobials for UTI are often not appropriate or suboptimal in the context of the subsequent laboratory analysis of urine by culture and microscopy. Additionally, the widely recommended first line empirical treatment is trimethoprim or nitrofurantoin [31], which were prescribed to only 37% of the patients. These guidelines do not contain the limitation included in other recommendations [7] to avoid trimethoprim when trimethoprim resistance levels exceed 20% or when trimethoprim was prescribed in the previous 3 months [32]. A previous publication in this region has included a caution regarding the use of trimethoprim because of the high levels observed [9]. The poor adherence to current recommendations for management of UTI is also described in a Spanish audit study [33]. Even though this study did not compare the prescription of the antimicrobial with the laboratory results, they found that 96% of the women with clinical criteria compatible with lower UTI received an antimicrobial and first line recommended treatment was prescribed to 32% of the patients. In a Dutch study of 470 patients with suspected UTI, half of the patients were prescribed first line antimicrobials and nearly 15% received a prescription for fluoroquinolones [34]. In our study, some practices showed clear preferences for recommended first line agents while others preferred fluoroquinolones. Levels of resistance to ciprofloxacin in *E.coli *increased in Ireland from 5.3 in 2002 to 8.3% in 2009 [9,35]. Similar tendencies have been observed in other countries [36-38] and some countries showed the proportion of ciprofloxacin resistant *E.coli *as high as 13%, which warrants against its use in uncomplicated UTI [39]. The reasons for the poor adherence to guidelines were studied in a qualitative study and the results suggested that the perceived lack of applicability to the practice population was the main barrier to implementing the guidelines [40].

In addition to overall (increased) prescribing of antimicrobials and its impact on resistance levels [41], practice variation in the use of antimicrobials has also shown to have an impact in an analysis in four UK administrations [42]. The relevance of practice variation for resistance levels has previously been described by us in a multilevel analysis of retrospective data from 72 practices over a 4.5 year period. In this analysis it was shown that the variation in levels of resistance (in uropathogenic *E.coli*) between practices was higher for ciprofloxacin than it was for trimethoprim and that both were associated with overall practice prescribing of the antimicrobial [35]. It has been suggested that it is likely that limitation of fluoroquinolone prescribing will curtail fluoroquinolone resistance levels [43] while limiting trimethoprim prescribing is less likely to influence the more established resistance to trimethoprim [44,45].

Empiric antimicrobial treatment was prescribed to 56% of the patients including 37% of patients without laboratory evidence of UTI. A surveillance study showed that more than 80% of the patients presenting with a suspected UTI in English general practice received an antimicrobial [46]. A comparable study from Germany in which similar inclusion criteria as described by us were used, also found 56% prescribing overall and 22% of the prescribing was for patients without any evidence of urinary tract infection [25]. It is clear that there remains scope for reductions in antimicrobial prescribing in general practice and symptomatic treatment of patients with suspected UTI might be an option. A Belgian study has shown that half of the patients were free of symptoms after 3 days of placebo [47] and a recent trial showed no difference between symptomatic treatment with ibuprofen or ciprofloxacin for uncomplicated UTI [48]. A study comparing different antimicrobial strategies with placebo in a large UTI trial showed slightly poorer results for the placebo group [49]. These results suggest that UTI is often a self-limiting disorder and symptomatic treatment of uncomplicated UTI deserves further research. However, when empiric treatment is preferred, preference should be given to nitrofurantoin in the absence of any specific contraindication. A recent review comparing different classes of antimicrobials for treatment of acute uncomplicated UTI in women found no differences between trimethoprim, fluoroquinolones, ß-lactam antibiotics and nitrofurantoin for the symptomatic cure of acute uncomplicated UTI [8]. Nitrofurantoin is an appropriate agent because of low resistance levels in the community and relatively low cost. Additionally, a recent analysis of recurrent infections showed that resistance to nitrofurantoin was generally low and once detected, decays relatively quickly [4]. There are theoretical reasons to believe that it may be less likely to select for antimicrobial resistance as it is concentrated in urine whereas other agents are distributed extensively in all body compartments including the gastrointestinal tract [50,51].

## Conclusion

The treatment of uncomplicated UTI was considered appropriate for 55% of the patients. Antimicrobial treatment was prescribed to 56% of all patients with a recommended first line treatment for 38% of these prescriptions. There appears to be considerable scope to reduce the frequency of antimicrobial prescribing as well as improve the quality of antimicrobial prescriptions for patients presenting to GPs with symptoms suggestive of UTI. When an antimicrobial is prescribed uniform use of nitrofurantoin (except when contraindicated) would reduce drug cost without affecting the appropriateness of treatment. Trimethoprim is not as appropriate as resistance levels of uropathogenic *E.coli *to trimethoprim in the study region are relatively high.

## Competing interests

The authors declare that they have no competing interests.

## Authors' contributions

AV set up and coordinated the study, analysed the results and drafted the manuscript. MC and AWM conceived of the study and critically revised the manuscript. BH acquired the data for the study and approved the final manuscript. KB has been involved in the conception of the study and has approved the final manuscript.

## Pre-publication history

The pre-publication history for this paper can be accessed here:

http://www.biomedcentral.com/1471-2296/12/108/prepub
